# Prospective Longitudinal Cohort of Argentinean Patients with Progressive Supranuclear Palsy: A Platform for Epidemiological and Translational Research

**DOI:** 10.1002/alz70860_105413

**Published:** 2025-12-23

**Authors:** Blas Couto, María Jose Angel Pinto, Maria‐Elena Avale, Tomas Falzone, Oscar Gershanik, Anabel Chade, Gabriel Mizraji, Maria Eugenia Gonzalez‐Toledo, Maria Peralta, Gonzalo Gomez‐Arevalo, Monica Mezmezian, Connie Marras, Gustavo Sevlever

**Affiliations:** ^1^ Laboratory of Progressive Neuromotor and Neurobehavioral Disorders at Instituto de Neurociencia Cognitiva y Traslacional (INCYT), CABA, Buenos Aires, Argentina; ^2^ Unidad de Movimientos Anormales, Institute of Neuroscience of the Fundacion Favaloro, CABA, Buenos Aires, Argentina; ^3^ Institute of Cognitive and Translational Neuroscience (INCyT) Favaloro‐INECO‐CONICET, Buenos Aires, Buenos Aires, Argentina; ^4^ Instituto Nacional de Geriatría, Santiago de Chile, Santiago, Chile; ^5^ Instituto de Investigaciones en Ingeniería genética y Biología Molecular (INGEBI‐CONICET), Buenos Aires, Argentina; ^6^ Instituto de Investigacion en Biomedicina de Buenos Aires. Partner Institute of the MaxPlank society. (IBioBA‐CONICET‐MPSP), Buenos Aires, Argentina; ^7^ IBCN (UBA‐CONICET), Facultad de Medicina, Universidad de Buenos Aires, Buenos Aires, Argentina; ^8^ Neurologia, Universidad Favaloro, CABA, Buenos Aires, Argentina; ^9^ Instituto de Neurología Cognitiva (INECO), CABA, Buenos Aires, Argentina; ^10^ Favaloro, Unidad de Movimientos Anormales, Buenos Aires, CABA, Argentina; ^11^ Clínica de Enfermedad de Parkinson y Movimientos Anormales, Departamento de Neurociencias, Hospital Universitario CEMIC, Centro de Educación Médica e Investigaciones Clínicas “Norberto Quirno”, BUenos Aires, CABA, Argentina; ^12^ Laboratorio de Neuropatología y Biología Molecular, Banco de cerebros, Fleni, Buenos Aires, CABA, Argentina; ^13^ University of Toronto, Toronto, ON, Canada; ^14^ Krembil Research Institute, University Health Network, Toronto, ON, Canada; ^15^ Fleni, CABA, Buenos Aires, Argentina

## Abstract

**Background:**

Progressive supranuclear palsy (PSP) and corticobasal syndrome (CBS) are two neurodegenerative disorders that manifest disabling postural instability, parkinsonism, cognitive impairment and falls with brain neurofibrillary tangle lesions containing 4‐repeat tau protein. The prevalence is 6/100,000 inhabitants but estimated only from descriptions made in North America, Europe or Japan. To describe a multicenter collaborative initiative for studying the epidemiology and tau biology of Argentinean patients with PSP and CBS, Consorcio Argentino de Investigación Traslacional en Tauopatias Primarias (CAITauP).

**Method:**

A prospective observational cohort of people with PSP (PwPSP) and CBS will be longitudinally studied. Deep clinical phenotyping, neurocognitive testing every 12 months, and biosamples collection at baseline are planned. Disease severity will be assessed every 6 months and history of environmental exposures will be assessed at baseline.

**Result:**

There were 50 patients enrolled with PSP (36), CBS (10), and undifferentiated parkinsonism with cognitive impairment (4). Female gender was 51%, mean age 74 years, with estimated disease duration of 5.5 years. Ethnicity was mostly Hispanic/Latino (78%), european descendant (28.5%), and assian (1.5%). Variant phenotypes of PSP were: 36% Richardson syndrome, 19%PSP‐Progressive gait freezing, 18% PSP‐corticobasal, 14% PSP‐parkinsonsm, 9% PSP‐Speech/languaje variant, 3% PSP‐pure postural inestability, and 1,5% pure PSP‐oculomotor. Disease severity measured by the PSP‐rating scale (PSPRS) was 54. Preliminary findings of the initially enrolled patients showed that more than 60% of enrolled patients have completed first visit and biosamples collection, and two patients underwent brain donation. Environmental exposures of a subsample are reported and compared to those of patients with Parkinson's disease.

**Conclusion:**

The CAITauP includes a comprehensive cohort of PwPSP in Argentina that has achieved progress in patient recruitment, deep clinical phenotyping and biobanking. Early findings highlight the feasibility of translational research in tauopathies in developing countries of South America. The consortium aims to address underrepresented groups of South American populations in ongoing research aimed to improve diagnosis, treatment, and to create trial‐ready cohorts that enhance translational research in PSP.

